# Germinated Pigmented Rice (*Oryza Sativa* L. cv. Superhongmi) Improves Glucose and Bone Metabolisms in Ovariectomized Rats

**DOI:** 10.3390/nu8100658

**Published:** 2016-10-21

**Authors:** Soo Im Chung, Su Noh Ryu, Mi Young Kang

**Affiliations:** 1 Department of Food Science and Nutrition, Brain Korea 21 Plus, Kyungpook National University, Daegu 41566, Korea; zizibe0312@nate.com; 2 Department of Agricultural Science, Korea National Open University, Seoul 03087, Korea; ryusn@knou.ac.kr

**Keywords:** pigmented rice, germination, Superhongmi, bone metabolism, glucose

## Abstract

The effect of germinated Superhongmi, a reddish brown pigmented rice cultivar, on the glucose profile and bone turnover in the postmenopausal-like model of ovariectomized rats was determined. The ovariectomized Sprague-Dawley rats were randomly divided into three dietary groups (*n* = 10): normal control diet (NC) and normal diet supplemented with non-germinated Superhongmi (SH) or germinated Superhongmi (GSH) rice powder. After eight weeks, the SH and GSH groups showed significantly lower body weight, glucose and insulin concentrations, levels of bone resorption markers and higher glycogen and 17-β-estradiol contents than the NC group. The glucose metabolism improved through modulation of adipokine production and glucose-regulating enzyme activities. The GSH rats exhibited a greater hypoglycemic effect and lower bone resorption than SH rats. These results demonstrate that germinated Superhongmi rice may potentially be useful in the prevention and management of postmenopausal hyperglycemia and bone turnover imbalance.

## 1. Introduction

Germination is considered as a simple, effective, and inexpensive method of improving the nutritional quality of rice [1]. The soaking of rice grains in water for a few days induces slight germination which causes an increase in nutrient bioavailability and absorption [2]. During germination, biochemical changes occur including the release of free and bound materials and the activation of dormant enzymes which break down large molecular substances, resulting in the generation of bioactive compounds and an increase in nutrients [3]. Germinated rice has been found to have higher amounts of bioactive compounds such as γ-oryzanol, γ-aminobutyric acid (GABA), tocopherols, and tocotrienols than non-germinated rice [4,5]. Moreover, it has been shown to possess strong antidiabetic, antihyperlipidemic, and antioxidative properties [1,6].

Pigmented rice cultivars with black, purple, red, or brown pericarp are known for their higher nutritional value and greater antioxidant potential than non-pigmented cultivars [7,8]. They contain high amounts of anthocyanins, phenolic compounds and bioactive components [9,10] and their consumption has been associated with a reduced risk of diabetes and cardiovascular disease [11]. Investigations on various pigmented cultivars revealed that ingestion of pigmented rice could improve the lipid and glucose profiles in mice, delay the starch and sugar absorption in rats, and suppress postprandial blood sugar elevation in human subjects [12,13].

Menopause, the permanent cessation of menstruation, promotes metabolic syndromes and increases the risk of diabetes, dyslipidemia, and obesity in women [14]. An elevation in the concentrations of glucose, insulin, cholesterol, and triglyceride has been observed in postmenopausal women relative to premenopausal ones [14,15,16]. Menopause is also believed to be associated with the pathogenesis of osteoporosis, a metabolic bone disorder characterized by enhanced bone fragility and increased fracture risk, in elderly women [17]. The rapid decrease of the ovarian hormone estrogen after menopause is regarded as the primary cause of these metabolic dysfunctions [14]. The surgical removal of ovaries, known as ovariectomy, mimics the estrogen-deficient condition in postmenopausal women. Hence, ovariectomized animal models are widely used in investigating the pathophysiological changes associated with menopause and in developing therapeutic strategies against menopause-induced metabolic disorders [18].

Superhongmi is a new pigmented rice cultivar with a reddish brown pericarp developed in Korea. Recent studies have shown that germinated Superhongmi rice has a strong antioxidant capacity and could improve the lipid metabolism in ovariectomized rats [19,20]. To further explore the therapeutic potential of Superhongmi rice against metabolic dysfunctions, particularly those caused by menopause, the present study investigated the effect of germinated Superhongmi rice on the glucose metabolism and bone turnover in the postmenopausal-like model of ovariectomized rats.

## 2. Materials and Methods

### 2.1. Rice Samples and Chemicals

Whole grains of Superhongmi rice were obtained from the department of Agricultural Science, Korea National Open University. They were grown from May to October 2014 in Dangjin, Chungcheongnam-do, South Korea. All chemicals and standards used in this study were of analytical grade and purchased from Merck KGaA (Darnstadt, Germany) and Sigma-Aldrich, Inc. (Steinhein, Germany).

### 2.2. Rice Germination

Dehusked whole rice grains were germinated following the method of Wu et al. [21] with slight modifications. The grains (50 g) were washed twice with distilled water to remove any dirt and placed evenly in a tray overlaid with cotton pads and cheesecloth. Distilled water (100 mL) was added and the whole tray was covered with a clean transparent plastic wrap with holes to allow for ventilation and incubated at 30 °C in an oven. The grains were regularly checked every 12 h to ensure there was no foul odor and fungal growth. After 72 h, the germinated rice grains, including the emerged radicles, were dried at 50 °C for 2 h, ground and pulverized (200–300 µm) using a grinding machine (HMF-3250S, Hanil Electronics, Seoul, South Korea), packed in hermetically sealed Ziploc plastic bags, and stored at −20 °C until further analysis. For the non-germinated samples, 50 g rice grains were washed, dried, ground (200–300 µm), and stored using the same method described above for the germinated grains. Both the germinated and non-germinated rice samples were analyzed for their bioactive compounds γ-oryzanol, GABA, phytic acid, tocols (tocopherols and tocotrienols), and policosanol, based on previously described methods [22,23,24,25,26] and for their proximate compositions using the methods of AOAC [27]. The results are shown in Table 1.

### 2.3. Animals and Diet

Thirty female ovariectomized Sprague-Dawley rats (10-week-old), weighing approximately 229 g each, were purchased from Central Laboratory Animal Inc. (Seoul, Korea). The animals were individually housed in a hanging stainless steel cage in a room (25 ± 2 °C, 50% relative humidity) with 12/12 h light-dark cycle and fed initially with a pelletized chow diet and distilled water ad libitum for 1 week. They were then randomly divided into three dietary groups (*n* = 10): normal control diet (NC) and NC diet supplemented with either 20% (w/w) non-germinated Superhongmi (SH) or germinated Superhongmi (GSH) rice powder. They were fed for 8 weeks and allowed free access to distilled water. The composition of the experimental diet (Table 2) was based on the AIN-93M diet [28]. At the end of the experimental period, the rats were anaesthetized with carbon dioxide by inhalation following a 12-h fast. The blood samples were drawn from the inferior vena cava into a heparin-coated tube and centrifuged at 1000× *g* for 15 min at 4 °C to obtain the plasma. The liver, heart, kidney, and white adipose tissues (perirenal and inguinal) were removed, rinsed with physiological saline, weighed, and stored at −70 °C until analysis. The current study protocol was approved by the Ethics Committee of Kyungpook National University for animal studies (approval No. 2015-0087).

### 2.4. Determination of Glucose Profile and Plasma Adipokine Levels

The levels of blood glucose and plasma insulin were determined using Accu-Chek Active Blood Glucose Test Strips (Roche Diagnostics, Berlin, Germany) and enzyme-linked immunosorbent assay (ELISA) kits (TMB Mouse Insulin ELISA kit, Shibayagi Co., Gunma, Japan), respectively. The hepatic glycogen level was determined using the anthrone-H_2_SO_4_ method with glucose as standard [29]. The homeostasis model assessment of insulin resistance (HOMA-IR) index was calculated using the equation described by Vogeser et al. [30]. The following plasma adipokines were analyzed using commercial assay kits: adiponectin (Shibayagi Co., Gunma, Japan), leptin (Cayman Chemical, Ann Arbor, MI, USA), resistin (B-Bridge International Inc., Santa Clara, CA, USA), and tumor necrosis factor (TNF)-α (Abcam, Cambridge, MA, USA).

### 2.5. Determination of Hepatic Glucose-Regulating Enzymes Activities

The liver tissue was homogenized in a buffer solution containing triethanolamine, EDTA, and dithiothreitol and centrifuged at 1000× *g* at 4 °C for 15 min [31]. The pellet was removed and the supernatant was centrifuged at 10,000× *g* at 4 °C for 15 min. The resulting precipitate served as the mitochondrial fraction and the supernatant was further centrifuged at 105,000× *g* at 4 °C for 1 h. The resulting precipitate and supernatant served as the microsome and cytosol fractions, respectively. The protein content was measured using the Bradford protein assay [32]. The phosphoenolpyruvate carboxykinase (PEPCK) activity was determined based on the method of Bentle and Lardy [33]. The absorbance of the assay mixture was measured at 340 nm. The glucokinase (GK) activity was measured following the method described by Davidson and Arion [34]. The reaction mixture was incubated at 37 °C for 10 min and the change in absorbance at 340 nm was recorded. The glucose-6-phosphatase (G6pase) activity was determined using the method of Alegre et al. [35]. The reaction mixture was incubated at 37 °C for 4 min and the change in absorbance at 340 nm was recorded. The enzyme activities were expressed as µmol/min/mg protein.

### 2.6. Measurement of Bone Metabolism Biochemical Markers

The levels of calcium and alkaline phosphatase (ALP) were measured using Ca and ALP assay kits (Cobas, Indianopolis, IN, USA), respectively. The levels of 17-β-estradiol, intact parathyroid hormone (PTH), osteocalcin, *N*-terminal telopeptide of type 1 collagen (NTx-1) and *C*-terminal telopeptide of type 1 collagen (CTx-1) were analyzed using commercial assay kits (MyBiosource Inc., San Diego, CA, USA).

### 2.7. Statistical Analysis

All data are presented as the mean ± standard error (SE). The data were evaluated by one-way ANOVA using a Statistical Package for Social Sciences software program version 19.0 (SPSS Inc., Chicago, IL, USA) and the differences between the means were assessed using Tukey’s test. An independent *t*-test was used to assess the difference between the germinated and non-germinated rice samples. Statistical significance was considered at *p* < 0.05.

## 3. Results

### 3.1. Body and Organ Weights

The final body weight markedly decreased in both SH (389 g) and GSH (374 g) groups relative to that of the control group (403 g) (Table 3). The feed intake and feed efficiency ratio were lowest in the GSH group and highest in the NC group. The white adipose tissue weight was lowest in GSH rats (8.56 g), followed by the SH group (9.04 g), then the NC group (10.26 g). The weights of liver and heart were significantly lower in the SH and GSH groups compared to that of the NC group.

### 3.2. Glucose Profile

As shown in Table 4, the final blood glucose level was lowest in the GSH group (5.04 nmol/L), followed by the SH group (5.61 nmol/L), then the NC group (6.98 nmol/L). The plasma insulin level was also lowest in the GSH group (3.39 mU/L) and highest in the NC group (4.93 mU/L). Accordingly, the HOMA-IR index was highest in the NC group (1.49), followed by the SH group (0.97), then the GSH group (0.78). Both the SH and GSH groups showed significantly higher hepatic glycogen level (149–153 mg/g) than the NC group (94.7 mg/g).

### 3.3. Plasma Adipokine Level

The adiponectin level was highest in the GSH group (0.71 ng/mL) and lowest in the NC group (0.26 ng/mL) (Table 4). On the other hand, the levels of resistin and TNF-α were lowest in the GSH group and highest in the NC group. No significant difference was found in the leptin level among the animal groups.

### 3.4. Hepatic Glucose-Regulating Enzymes Activities

The hepatic PEPCK and G6pase activities were lowest in GSH rats and highest in the NC group (Table 4). Both the SH and GSH rats exhibited significantly higher GK activity (2.89–2.98 μmol/min/mg protein) than the control ones (1.62 μmol/min/mg protein). The GK to G6pase ratio was highest in the GSH group (0.06), followed by the SH group (0.04), then the NC group (0.02).

### 3.5. Biochemical Markers of Bone Metabolism

The GSH group showed significantly higher levels of 17-β-estradiol (0.87 ng/mL) and lower levels of intact PTH (18.05 pg/mL), NTx-1 (121.44 nmol/L), and CTx-1 (13.29 nmol/L) than the NC and SH groups (Table 5). No significant difference was found in the calcium and osteocalcin contents among the groups. The ALP level, on the other hand, was below 0.50 μg/L in all groups.

## 4. Discussion

Ovarian hormone deficiency resulting from menopause or ovariectomy increases the risk of diabetes, obesity, dyslipidemia, and osteoporosis [14,17,36]. The present study analyzed the effect of germinated Superhongmi rice, a reddish-brown pigmented cultivar, on the glucose and bone metabolisms in the postmenopausal-like model of ovariectomized rats. Results showed that diet supplementation of germinated and non-germinated Superhongmi rice powder significantly decreased the body weight gain, amount of body fat, blood glucose level, and plasma insulin concentrations and increased the hepatic glycogen level in ovariectomized rats. Both the SH and GSH animal groups also exhibited a markedly lower HOMA-IR index—an indicator of insulin resistance—than the control group, suggesting an increase in the insulin sensitivity in these animals. Studies in the past have also shown that pigmented rice could lower the body weight gain and improve the glucose metabolism in both laboratory animals and human subjects [12,13]. Between the two Superhongmi rice-fed groups, the GSH rats exhibited a greater body weight-lowering effect and hypoglycemic activity than the SH group. Germinated rice, especially pigmented cultivar, contains substantially higher amounts of bioactive compounds than non-germinated rice [4,5,19]. In the present study, γ-oryzanol, GABA, phytic acid, tocols, and policosanol were significantly higher in a germinated rice sample than that of the non-germinated one. γ-oryzanol, GABA, and phytic acid have hypolipidemic, hypoglycemic, and anti-obesity effects [4,7,37,38,39]. The tocols and policosanol possess antioxidative and antidiabetic property [4,40,41]. Hence, the strong hypoglycemic activity observed in GSH rats relative to the SH group is probably due to the increased amounts of bioactives in the germinated Superhongmi rice. This increase in the bioactive content is caused by the breaking down of the cell wall during germination, releasing the free and bound materials, and the activation of dormant enzymes associated with the synthesis of bioactive compounds [3].

The metabolism of glucose is influenced by adipokines and glucose-regulating enzymes. The ovariectomized rats fed with germinated Superhongmi rice powder showed the lowest resistin and TNF-α levels and highest adiponectin concentration. They also exhibited the lowest PEPCK and G6pase activities and highest GK activity and GK/G6pase ratio. The adipokines resistin and TNF-α regulate the lipid and glucose metabolisms and their elevated levels have been associated with the progression of obesity and diabetes [42,43,44]. The adiponectin, on the other hand, induces insulin-sensitizing effects and its enhanced expression has been shown to improve insulin sensitivity and glucose tolerance while its deficiency could induce insulin resistance [42]. An elevated level of adiponectin has also been reported to protect postmenopausal women against the development of diabetes [45]. The PEPCK, GK, and G6pase are major enzymes associated with glucose metabolism, wherein PEPCK and G6pase are involved in the regulation of gluconeogenesis and hepatic glucose output and an increase in their activities could result in an increased production of glucose [46,47]. The GK enzyme, on the other hand, is involved in glucose homeostasis and its enhanced activity has been associated with an increased glycogen level and reduced blood glucose content [48]. The GK/G6pase ratio, which reflects the balance between glucose uptake and output, was highest in GSH rats, indicating an enhanced glucose metabolism in these animals. Thus, the increase in adiponectin level and GK activity and the reduction in resistin and TNF-α concentrations and PEPCK and G6pase activities are possibly responsible for the improved glucose profile found in the rice-fed ovariectomized rats, particularly the GSH group.

Menopause and ovariectomy cause metabolic dysfunctions due to the rapid decrease of the estrogen hormone [14]. In the present study, the GSH rats showed significantly higher amount of 17-β-estradiol—the most potent form of estrogen—than the control group, suggesting that the germinated Superhongmi rice was able to inhibit the ovariectomy-induced reduction of estrogen level in these animals. Estrogen plays a central role in the regulation of bone metabolism, and administration of 17-β-estradiol has been reported to decrease the rate of bone turnover and prevent bone loss in postmenopausal women [49,50,51]. The ovariectomized rats fed with germinated Superhongmi rice also exhibited relatively low levels of intact PTH, NTx-1, and CTx-1, which are biochemical markers of bone resorption, indicating a reduced bone turnover in the GSH group. Increased bone resorption and imbalanced bone turnover are considered the main cause of the rapid rate of bone loss and enhanced risk of bone fracture in postmenopausal women [51,52]. Rice cultivars with colored pericarp, such as Superhongmi, are rich in antioxidant compounds such as anthocyanins, tocols, γ-oryzanol, and phytic acid [53] and germination could further increase the amount of these antioxidant compounds. Germinated Superhongmi rice has been previously reported to contain a substantial amount of antioxidant compounds and to have a strong antioxidant capacity [19]. Past investigations revealed that dietary antioxidants could prevent bone loss in postmenopausal women and ovariectomized animals and may be useful in the prevention and treatment of osteoporosis [54,55]. Since oxidative stress plays a central role in the pathogenesis of osteoporosis [56,57], the antioxidant compounds present in germinated Superhongmi rice may have been partly responsible for the improved bone metabolism observed in GSH rats.

## 5. Conclusions

The pigmented rice Superhongmi significantly reduced the body weight gain, glucose level, insulin concentration, and bone turnover in the postmenopausal-like model of ovariectomized rats through a mechanism involving the regulation of adipokine production and modulation of glucose-regulating enzyme activities. Germination for 72 h further enhanced the hypoglycemic effect and bone metabolism-improving action of this pigmented rice cultivar which may have been due to the increased amounts of various bioactive compounds such as GABA, γ-oryzanol, and tocols. Germinated Superhongmi rice may be beneficial as a functional food with therapeutic potential against menopause-induced hyperglycemia and bone turnover imbalance.

## Figures and Tables

**Table 1:** Bioactive components and proximate composition of Superhongmi rice powder.

Bioactive Compound	Non-Germinated	Germinated
γ-Oryzanol (mg/100 g rice)	33.21 ± 2.66	51.96 ± 1.99 ^1,^*
GABA (mg/100 g rice)	98.54 ± 3.96	1102.02 ± 11.63 *
Phytic acid (mg/100 g rice)	2.01 ± 0.09	4.02 ± 0.14 *
Tocols (μg/100 g rice)	133.69 ± 8.62	256.79 ± 6.98 *
Policosanol (mg/100 g rice)	21.69 ± 1.02	26.51 ± 1.24 *
Proximate composition (% dry basis)		
Carbohydrates	76.58 ± 0.91 *	53.92 ± 0.98
Crude protein	7.11 ± 0.12 *	5.71 ± 0.41
Crude fat	2.31 ± 0.19	3.58 ± 0.17 *
Crude ash	1.34 ± 0.04 *	1.11 ± 0.02
Moisture	12.66 ± 0.32	35.68 ± 0.61 *

^1^ Values are means ± standard error (*n* = 3); * indicates significant difference (*p* < 0.05) between germinated and non-germinated samples.

**Table 2:** Composition of experimental diets (%).

	NC ^1^	SH	GSH
Casein	14.0	12.4	12.2
Sucrose	10.0	10.0	10.0
Dextrose	15.5	15.5	15.5
Corn starch	46.6	28.7	29.1
Cellulose	5.00	5.00	5.00
Soybean oil	4.00	3.50	3.24
Mineral mix	3.50	3.50	3.50
Vitamin mix	1.00	1.00	1.00
l-Cystine	0.18	0.18	0.18
Choline bitartrate	0.25	0.25	0.25
Non-germinated rice	-	20.0	-
Germinated rice	-	-	20.0
Total	100	100	100
Kcal	380	380	380

^1^ NC, normal control diet (AIN-93M); SH, normal diet + non-germinated Superhongmi rice powder; GSH, normal diet + germinated Superhongmi rice powder.

**Table 3:** Body weight gain and weights of organs and adipose tissue in ovariectomized rats fed with germinated Superhongmi rice powder.

Parameter	NC	SH	GSH
Initial weight (g)	229.24 ± 1.25	228.32 ± 1.18	228.14 ± 0.79
Final weight (g)	402.65 ± 5.33 ^c^	388.69 ± 4.92 ^b^	374.25 ± 5.41 ^a^
Weight gain (g)	174.68 ± 5.63 ^c^	160.24 ± 4.72 ^b^	148.32 ± 3.30 ^a^
Feed intake (g/week)	181.58 ± 4.32 ^c^	162.25 ± 3.20 ^b^	149.44 ± 3.41 ^a^
Feed efficiency ratio	0.16 ± 0.00 ^c^	0.14 ± 0.00 ^b^	0.12 ± 0.00 ^a^
White adipose tissue weight (g)	10.26 ± 0.19 ^c^	9.04 ± 0.19 ^b^	8.56 ± 0.12 ^a^
Organ weight (g)			
Liver	2.88 ± 0.01 ^c^	2.57 ± 0.02 ^b^	2.50 ± 0.01 ^a^
Heart	0.26± 0.01 ^b^	0.23 ± 0.01 ^a^	0.22 ± 0.01 ^a^
Kidney	0.40 ± 0.01	0.39 ± 0.02	0.39 ± 0.04

^a−c^ Values are means ± SE (*n* = 10). Means in the same row not sharing a common superscript are significantly different at *p* < 0.05. NC, normal control diet (AIN-93M); SH, normal diet + non-germinated Superhongmi; GSH, normal diet + germinated Superhongmi rice.

**Table 4:** Glucose profile, adipokine level, and glucose-regulating enzyme activity in ovariectomized rats fed with germinated Superhongmi rice powder.

Parameter	NC	SH	GSH
Initial blood glucose (mmol/L)	4.98 ± 0.02	5.01 ± 0.02	5.08 ± 0.02
Final blood glucose (mmol/L)	6.98 ± 0.05 ^c^	5.61 ± 0.03 ^b^	5.04 ± 0.03 ^a^
Plasma insulin (mU/L)	4.93 ± 0.03 ^c^	3.91 ± 0.05 ^b^	3.39 ± 0.01 ^a^
Hepatic glycogen (mg/g liver)	94.68 ± 2.26 ^a^	149.25 ± 2.78 ^b^	152.88 ± 3.07 ^b^
HOMA-IR index	1.49 ± 0.00 ^c^	0.97 ± 0.02 ^b^	0.78 ± 0.00 ^a^
Plasma adipokine			
Adiponectin (ng/mL)	0.26 ± 0.03 ^a^	0.48 ± 0.03 ^b^	0.71 ± 0.06 ^c^
Leptin (ng/mL)	3.76 ± 0.27	3.32 ± 0.33	3.36 ± 0.26
Resistin (ng/mL)	32.55 ± 0.12 ^c^	22.88 ± 1.43 ^b^	18.25 ± 1.05 ^a^
TNF-α (µg/mL)	9.58 ± 0.81 ^c^	7.25 ± 0.58 ^b^	4.51 ± 0.12 ^a^
*Hepatic glucose-regulating enzymes (µmol/min/mg protein)*			
PEPCK	3.74 ± 0.87 ^c^	2.98 ± 0.52 ^b^	1.18 ± 0.41 ^a^
GK	1.62 ± 0.13 ^a^	2.89 ± 0.19 ^b^	2.98 ± 0.22 ^b^
G6pase	76.95 ± 1.32 ^c^	68.33 ± 1.47 ^b^	47.58 ± 1.51 ^a^
GK/G6pase ratio	0.02 ± 0.00 ^a^	0.04 ± 0.00 ^b^	0.06 ± 0.00 ^c^

^a−c^ Values are means ± SE (*n* = 10). Means in the same row not sharing a common superscript are significantly different at *p* < 0.05. NC, normal control diet (AIN-93M); SH, normal diet + non-germinated Superhongmi rice; GSH, normal diet + germinated Superhongmi rice; HOMA-IR, homeostasis model of insulin resistance = (fasting insulin × fasting glucose)/22.5; TNF, tumor necrosis factor; PEPCK, phosphoenolpyruvate carboxynase; GK, glucokinase; G6pase, glucose-6-phosphatase.

**Table 5:** Biochemical markers of bone metabolism in ovariectomized rats fed with germinated Superhongmi rice powder.

	NC	SH	GSH
17-β-estradiol (ng/mL)	0.47 ± 0.03 ^a^	0.52 ± 0.02 ^a^	0.87 ± 0.05 ^b^
Intact PTH (pg/mL)	22.58 ± 0.63 ^b^	21.22 ± 1.02 ^b^	18.05 ± 0.57 ^a^
Calcium (mg/dL)	9.65 ± 0.53	10.68 ± 0.43	10.58 ± 0.58
Osteocalcin (ng/mL)	13.55 ± 1.23	13.16 ± 0.73	12.57 ± 0.54
Alkaline phosphatase (µg/L)	<0.50 ± 0.00	<0.50 ± 0.00	<0.50 ± 0.00
NTx-1 (nmol/L)	181.58 ± 2.37 ^c^	145.25 ± 1.23 ^b^	121.44 ± 3.45 ^a^
CTx-1 (nmol/mL)	23.71 ± 0.85 ^c^	18.69 ± 0.65 ^b^	13.29 ± 1.58 ^a^

^a−c^ Values are means ± SE (*n* = 10). Means in the same row not sharing a common superscript are significantly different at *p* < 0.05. NC, normal control diet (AIN-93M); SH, normal diet + non-germinated Superhongmi rice; GSH, normal diet + germinated Superhongmi rice; PTH, parathyroid hormone; NTx-1, *N*-terminal telopeptide of type 1 collagen; CTx-1, *C*-terminal telopeptide of type 1 collagen.
